# Brexucabtagene autoleucel for relapsed or refractory mantle cell lymphoma in the United Kingdom: A real‐world intention‐to‐treat analysis

**DOI:** 10.1002/hem3.87

**Published:** 2024-06-13

**Authors:** Maeve A. O'Reilly, William Wilson, David Burns, Andrea Kuhnl, Frances Seymour, Ben Uttenthal, Caroline Besley, Rajesh Alajangi, Thomas Creasey, Shankara Paneesha, Johnathon Elliot, Carlos Gonzalez Arias, Sunil Iyengar, Matthew R. Wilson, Alison Delaney, Lourdes Rubio, Jonathan Lambert, Khalil Begg, Stephen Boyle, Kathleen P. L. Cheok, Graham P. Collins, Claire Roddie, Rod Johnson, Robin Sanderson

**Affiliations:** ^1^ University College London Hospital London UK; ^2^ University College London Cancer Institute London UK; ^3^ University College London and CRUK Cancer Trials Centre London UK; ^4^ University Hospital Birmingham Birmingham UK; ^5^ Kings College Hospital London UK; ^6^ Leeds Teaching Hospital Leeds UK; ^7^ Cambridge University Hospital Cambridge UK; ^8^ University Hospital Bristol Bristol UK; ^9^ Newcastle upon Tyne Hospitals Newcastle upon Tyne UK; ^10^ The Christie NHS foundation Trust Manchester UK; ^11^ The Royal Marsden Hospital London UK; ^12^ Beatson West of Scotland Cancer Centre Glasgow UK; ^13^ Sheffield Teaching Hospital Sheffield UK; ^14^ Manchester Royal Infirmary Manchester UK; ^15^ Oxford University Hospital Oxford UK

## Abstract

Brexucabtagene autoleucel (brexu‐cel) is an autologous CD19 CAR T‐cell product, approved for relapsed/refractory (r/r) mantle cell lymphoma (MCL). In ZUMA‐2, brexu‐cel demonstrated impressive responses in patients failing ≥2 lines, including a bruton's tyrosine kinase inhibitor, with an overall and complete response rate of 93% and 67%, respectively. Here, we report our real‐world intention‐to‐treat (ITT) outcomes for brexu‐cel in consecutive, prospectively approved patients, from 12 institutions in the United Kingdom between February 2021 and June 2023, with a focus on feasibility, efficacy, and tolerability. Of 119 approved, 104 underwent leukapheresis and 83 received a brexu‐cel infusion. Progressive disease (PD) and/or manufacturing (MF) were the most common reasons for failure to reach harvest and/or infusion. For infused patients, best overall and complete response rates were 87% and 81%, respectively. At a median follow‐up of 13.3 months, median progression‐free survival (PFS) for infused patients was 21 months (10.1–NA) with a 6‐ and 12‐month PFS of 82% (95% confidence interval [CI], 71–89) and 62% (95% CI, 49–73), respectively. ≥Grade 3 cytokine release syndrome and neurotoxicity occurred in 12% and 22%, respectively. On multivariate analysis, inferior PFS was associated with male sex, bulky disease, ECOG PS > 1 and previous MF. Cumulative incidence of non‐relapse mortality (NRM) was 6%, 15%, and 25% at 6, 12, and 24 months, respectively, and mostly attributable to infection. Outcomes for infused patients in the UK are comparable to ZUMA‐2 and other real‐world reports. However, ITT analysis highlights a significant dropout due to PD and/or MF. NRM events warrant further attention.

## INTRODUCTION

Mantle cell lymphoma (MCL), accounting for 3%–6% of non‐Hodgkin lymphoma (NHL),^1^ is a rare disease marked by significant clinical and pathological heterogeneity.^2^ While a subset of patients, particularly those with leukemic, non‐nodal variant, may initially exhibit features of indolent disease, MCL is usually aggressive with the majority of patients eventually requiring treatment. Disease risk profile is guided by factors such as the MCL international prognostic index (MIPI),^3^ response to frontline therapy, proliferation index (Ki‐67%),^4^ blastoid/pleomorphic subtypes^5^ and genetic aberrations such as TP53.^6^ Survival outcomes are increasingly poor with successive relapses,^7^ particularly after failure of a BTKi with a median overall survival (OS) of 1.4–11 months.^8^, ^9^, ^10^, ^11^ Chimeric antigen receptor (CAR) T‐cell therapy represents a new therapeutic option for eligible patients with relapsed refractory (r/r) MCL. The Phase 2 nonrandomised ZUMA‐2 study demonstrated a high initial overall response rate (ORR) of 93% with a complete response (CR) rate of 67% with brexucabtagene autoleucel (brexu‐cel) in r/r MCL.^12^ At a median follow‐up of 35.6 months, median progression‐free survival (PFS) and overall survival (OS) were 25.8 and 46.6 months, respectively and 37% of patients were in remission without further therapy.^12^, ^13^ In December 2020 the European Medicines Agency (EMA) granted conditional marketing authorization for brexu‐cel (Kite Gilead) in r/r MCL after ≥2 lines of therapy, including a bruton's tyrosine kinase inhibitor (BTKi). For the most part, real‐world outcomes post‐brexu‐cel infusion mirror the treatment response and survival outcomes achieved in ZUMA‐2, albeit with shorter follow‐up.^14^, ^15^, ^16^ Disease‐specific features associated with an inferior PFS include high simplified MIPI score,^15^, ^16^ Ki‐67 ≥ 50%, complex karyotype, *TP53* aberration, and blastoid/pleomorphic variant.^16^ However, rates of non‐relapse mortality (NRM) are significantly higher in the real‐world relative to ZUMA‐2 (3%), ranging from 9% to 15%,^15^, ^16^, ^17^ with a signal for late fatal infectious events. Identification of those most likely to benefit and an understanding of late toxicities are key to appropriate patient selection and tailored care post‐infusion.

Herein, we present our intention‐to‐treat (ITT) real‐world outcomes for brexu‐cel in the United Kingdom (UK) with a focus on feasibility, efficacy and tolerability in those with r/r MCL after a BTKi.

## METHODS

### Patients

Consecutive patients with r/r MCL, prospectively approved by the National CAR T Clinical Panel (NCCP), from 12 CAR T centres in England (or Scottish equivalent) between February 2021 and June 2023 were included (Table S1) (intention‐to‐treat cohort). The UK NCCP approval process has been previously described^18^ and data were extracted retrospectively from electronic records as part of a national service evaluation. Eligibility for treatment required ECOG PS of 0 or 1, absence of active central nervous system (CNS) disease and ≥2 lines of therapy including prior BTKi^19^ (Table S2). Organ function requirements were at the discretion of the treating centre.

### Definitions

Pre‐apheresis bridging therapy (BT) was defined as any lymphoma therapy delivered between NCCP approval and T‐cell apheresis. Post‐apheresis BT was defined as any lymphoma therapy delivered between T‐cell apheresis and admission for CAR T. All BT was administered at discretion of the treating physician. Manufacturing failure (MF) was defined as failure of the leukapheresis material to successfully yield a CAR T cell product that could be requested by the CAR T physician. Out of specification (OOS) products were defined as those that failed to reach release specifications but could be requested and infused at physician discretion. Lymphodepletion (LD) with fludarabine and cyclophosphamide was administered as per manufacturers' instructions. Cytokine release syndrome (CRS) and immune effector cell‐associated neurotoxicity syndrome (ICANS) were graded according to American Society for Transplantation and Cellular Therapy (ASTCT) consensus guidelines.^20^ Toxicity management strategies were at the discretion of the treating centre. Infection was defined as positive microbiology, virology, histopathology, and/or radiological findings as determined by the treating physician and considered alongside clinical symptoms. Culture‐negative neutropenic fever was excluded. Grades of infection were categorized as mild (no treatment or oral antibiotics), severe (requiring intravenous therapy), or life‐threatening (symptoms such as hemodynamic instability or requiring organ support).^21^, ^22^ Cumulative steroid dose was reported as dexamethasone equivalent (mg). Response to CAR‐T therapy was determined locally (Lugano classification 2014).^23^

### Statistical analyses

All analyses, graphs, and figures were performed and generated using Stata version 18.0, respectively. Progression‐free and overall survival were calculated separately from approval, apheresis, and infusion and were analysed using Kaplan–Meier survival analysis. Patients who did not have an event were censored at the date last seen. Cox proportional hazards models were used to estimate hazard ratios (HRs) and corresponding 95% confidence intervals (CIs) in both univariate and multivariable analysis (UVA; MVA). To avoid overfitting, MVA utilised a backward stepwise selection method (*p* = 0.05 for inclusion). NRM, measured from infusion until death without progression (disease progression treated as competing event), was analysed using a competing risks regression model by the method of Fine and Gray to estimate subhazard ratios (SHRs) and corresponding 95% CIs. Odds ratios, corresponding 95% CIs, and *p*‐values were estimated using logistic regression. Fisher's exact tests were used when the odds ratio could not be calculated. No adjustments have been made to account for multiple testing.

## RESULTS

### Patient characteristics

Baseline demographics of approved (*n* = 119), harvested (*n* = 104), and infused (*n* = 83) patients are summarized in Table 1. For infused patients, median age was 68 years (range 41–78) with a male predominance (60/83, 72%). Median number of prior lines was 2 (range 2–7); 35% and 17% had received a previous autologous and allogeneic stem cell transplant, respectively. Progression of disease within 24 months of front‐line therapy (POD24) was noted in 55% (45/82); 30% and 11% were BTKi‐refractory and refractory to all lines, respectively. Where data were available, 38% (21/55) had blastoid or pleomorphic disease, 38% (15/39) had a *TP53* mutation, 76% (35/46) had Ki‐67 ≥ 30% and 45% (31/69) had a high‐risk simplified MIPI score.

**Table 1:** Baseline characteristics of mantle cell lymphoma patients.

	Characteristic	Approved *N* = 119	Harvested *N* = 104	Infused *N* = 83
**Age, median (range)**		68 (41–80)	67.5 (41–78)	68 (41–78)
**Sex**				
	Female	32 (27%)	28 (27%)	23 (28%)
	Male	87 (73%)	76 (73%)	60 (72%)
**Prior lines, median (range)**		2 (2–7)	2 (2–7)	2 (2–7)
**Previous ASCT**				
	No	79 (66%)	69 (66%)	54 (65%)
	Yes	40 (34%)	35 (34%)	29 (35%)
**Previous Allo‐SCT**				
	No	104 (87%)	89 (86%)	69 (83%)
	Yes	15 (13%)	15 (14%)	14 (17%)
**POD24**				
	No	50 (43%)	42 (41%)	37 (45%)
	Yes	67 (57%)	61 (59%)	45 (55%)
	Unknown	2	1	1
**Refractory to all lines**				
	No	105 (88%)	92 (88%)	74 (89%)
	Yes	14 (12%)	12 (12%)	9 (11%)
**Ibrutinib refractory**				
	No	82 (70%)	72 (71%)	57 (70%)
	Yes	35 (30%)	30 (29%)	25 (30%)
	Unknown	2	2	1
**Ibrutinib intolerant**				
	No	111 (93%)	98 (94%)	78 (94%)
	Yes	8 (7%)	6 (6%)	5 (6%)
**History of CNS involvement**				
	No	116 (97%)	102 (98%)	82 (99%)
	Yes	3 (3%)	2 (2%)	1 (1%)
**ECOG PS at submission**				
	0	42 (35%)	37 (36%)	33 (40%)
	1	77 (65%)	67 (64%)	50 (60%)
**Most recent bendamustine dose**				
	<6 months	12 (11%)	12 (12%)	10 (12%)
	6–24 months	15 (14%)	15 (15%)	9 (11%)
	>24 months	15 (14%)	15 (15%)	14 (17%)
	None	65 (61%)	61 (59%)	49 (60%)
	Unknown	12	1	1
**sMIPI at submission**				
	Low	23 (23%)	20 (23%)	15 (22%)
	Intermediate	31 (31%)	28 (32%)	23 (33%)
	High	47 (47%)	40 (45%)	31 (45%)
	Unknown	18	16	14
**Ki‐67 at submission**				
	<30%	14 (22%)	13 (22%)	11 (24%)
	≥30	49 (78%)	46 (78%)	35 (76%)
	Unknown	56	45	37
**Morphological subtype at submission**				
	Blastoid	29 (37%)	26 (37%)	18 (33%)
	Classical^a^	46 (58%)	41 (58%)	34 (62%)
	Pleomorphic	4 (5%)	3 (4%)	3 (5%)
	Unknown	40	34	28
**TP53 aberration**				
	No aberration	28 (47%)	24 (49%)	22 (55%)
	TP53 aberration	31 (53%)	25 (51%)	18 (45%)
	Unknown	60	55	43
**TP53 mutation**				
	No TP53 mutation	35 (62%)	29 (63%)	24 (62%)
	TP53 mutation	21 (38%)	17 (37%)	15 (38%)
	Unknown	63	58	44
**Stage at submission**				
	I	1 (1%)	1 (1%)	1 (1%)
	II	11 (9%)	10 (10%)	10 (12%)
	III	10 (8%)	9 (9%)	8 (10%)
	IV	96 (81%)	83 (81%)	64 (77%)
	Unknown	1	1	0
**Bulk (>5 cm) at submission**				
	No	78 (66%)	66 (63%)	54 (65%)
	Yes	41 (34%)	38 (37%)	29 (35%)
**LDH at submission, median (range)**		231 (105–3209)	228 (105–2233)	227 (120–2233)
**EN sites at submission**				
	0	29 (24%)	26 (25%)	24 (29%)
	1	50 (42%)	42 (40%)	30 (36%)
	2	25 (21%)	24 (23%)	20 (24%)
	3	13 (11%)	12 (12%)	9 (11%)
	4	2 (2%)	0 (0%)	0 (0%)

Abbreviations: ASCT, autologous stem cell transplant; CNS, central nervous system; ECOG PS, Eastern Cooperative Oncology Group performance status; EN, extra‐nodal; LDH, lactate dehydrogenase; POD24, Progression of disease within 24 months of first‐line therapy; sMIPI, simplified MCL international prognostic index.

^a^ Leukaemic non‐nodal with classical morphology included in approved (*n* = 6), harvested (*n* = 5), and infused (*n* = 4) patients.

### Failure to reach cell harvest and/or infusion

**Figure 1:**
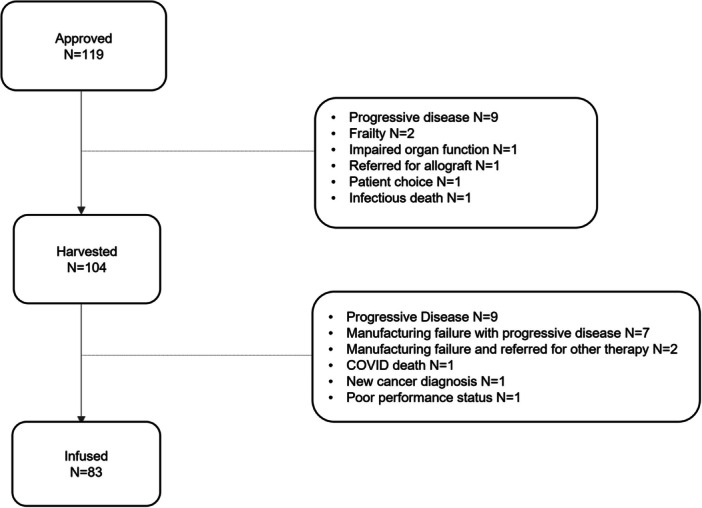
Flowchart of mantle cell lymphoma patients approved, harvested, and infused with brexu‐cel February 2021–June 23.

Seventy percent and 80% of approved and harvested patients, respectively were infused (Figure 1). Progressive disease (PD) was the most common reason for failure to reach cell harvest (9/15, 60%) and cell infusion (18/36, 50%). Where PD was cited as the primary reason for drop out, factors associated with higher rates of drop out included younger age and LDH > upper limit of normal (ULN). ECOG PS > 0 and the presence of extra‐nodal disease trended toward significance (Table S3). Other disease‐specific features such as *TP53* mutation and blastoid disease were not found to be significant.

MF with subsequent PD accounted for failure to reach cell infusion in 7/21 cases of drop out post‐T‐cell harvest (33%). MF occurred in 17/104 harvests (16.3%), with two MFs in two cases. Seven patients (41.2%) were not re‐harvested due to PD, one was referred for allograft and one was enrolled on a clinical trial. The remaining eight patients (47%) were infused after a second successful manufacture. On UVA, patients with circulating disease at T‐cell harvest were at higher risk of MF (odds ratio [OR]: 2.90, 95% CI 0.95–8.84, *p* = 0.06), particularly those with high burden in the peripheral blood. Of those with a lymphocyte count of >30 × 10^9^/L in peripheral blood on the day of harvest (*n* = 6), 75% (*n* = 4) had a failed first manufacture. Those with LDH > ULN had four times the odds of having a failed manufacture (OR: 4.01 (95% CI: 1.18–13.61, *p* = 0.03) (Table S4). There was no association between MF and CD3^+^ cell count in peripheral blood, total CD3^+^ cells harvested, number of lines of therapy, prior autograft/allograft, use of pre‐apheresis BT or Bendamustine exposure/timing (Table S4). Contrary to drop out due to PD which remained static between 2021 and 2023, the frequency of MF decreased over time (23% of harvests 2021 and 11% of harvests 2022) with no MFs at data cut‐off in 2023. Two OOS products were reported, one with a lower transduction efficiency (infused) and one with a visible particle on inspection of final product (re‐harvested).

### BT

Of approved patients, pre‐apheresis BT was administered to 56% (66/119) after approval and before T‐cell harvest (at physician discretion). In 49% (32/66), this therapy consisted of continuation of BTKi and/or steroids and/or Rituximab; the remainder receiving mostly R‐chemoimmunotherapy (Table S5). Ninety percent (94/104) of patients received BT after T‐cell harvest (Table S5). Forty‐six percent and 26% were bridged with chemoimmunotherapy and targeted therapy alone, respectively. Nineteen percent (18/94) were bridged with more than one modality, either concomitantly or sequentially. Bendamustine‐based regimens were the most commonly employed. Response to BT was assessed in 97% (91/94) with an ORR of 41% (11% CR and 30% PR). Median time from approval to harvest and harvest to infusion was 15 days (interquartile range [IQR]: 10–24) and 36 days (IQR: 33–43) respectively.

### Toxicity

Any grade CRS or ICANS occurred in 93% (≥grade 3 12%) and 55% (≥grade 3 22%), respectively. Median day of onset and duration for CRS and ICANS was Day 3 (range 0–10) and 5 days (range 1–11) and Day 8 (range 1–18) and 4 days (range 0–64), respectively. One grade 5 CRS and no grade 5 ICANS events were reported. Eighty percent and 58% received tocilizumab and steroids for toxicity management, respectively. Anakinra (*n* = 14, 17%) and siltuximab (*n* = 1, 1%) were also used as adjunctive agents for the management of ICANS. Factors pre‐LD associated with ≥grade 3 CRS on UVA included POD24, ≥3 extra‐nodal (EN) sites, bulk and ECOG PS > 1 (Table S6). No factors associated with ≥grade 3 ICANS were identified. Twenty‐nine (35%) patients developed a total of 35 infections within 1 month of infusion with a median of one infection per patient (range 1–3). 30/35 infections (86%) were deemed severe or life‐threatening, of which 83% (25/30) were bacterial in origin (Figure S1). Twenty‐seven percent (22/83) of patients required admission to an Intensive Care Unit (ICU) during their initial inpatient stay (ICANS *n* = 11, CRS+/− sepsis *n* = 8, observation of high‐risk disease site (airway/cardiac) *n* = 3). Grade 3/4 neutropenia and thrombocytopenia were reported in 59% and 60% at month 1 and 25% and 31% at month 3, respectively (Table 2).

**Table 2:** Toxicity profile of brexu‐cel and toxicity management strategies.

Infused patients *N* = 83	**CRS**	**ICANS**
Any grade	77 (93%)	46 (55%)
Maximum grade		
1	28 (34%)	17 (20%)
2	39 (47%)	10 (12%)
3	9 (11%)	17 (20%)
4	0	2 (2%)
5	1 (1%)	0
Days to onset, median (range)	3 (0–10)	8 (1–18)
Duration in days, median (range)	5 (1–11)	4 (0–64)

	CRS and/or ICANS
Steroid use	48 (58%)
Total cumulative steroid dose, median (range)	195 (10–1416)
Tocilizumab	66 (80%)
Anakinra	14 (17%)
Anakinra duration in days, median (range)	11 (3–30)
Siltuximab	1 (1%)

	Other toxicity
Any infection within 1 month of infusion (patients, *n*)	29 (35%)
Severe/life‐threatening infection within 1 month of infusion	25 (30%)
Intensive Care Unit Admission	22 (27%)
Non‐relapse mortality	12/83 (14.5%)
Cytopenia^a^	
G3/4 neutropenia at 1 month post‐infusion	48/81 (59%)
G3/4 thrombocytopenia at 1‐month post‐infusion	49/81 (60%)
G3/4 neutropenia at 3 months post‐infusion	17/67 (25%)
G3/4 thrombocytopenia at 3 months post‐infusion	21/67 (31%)

Abbreviations: CRS, cytokine release syndrome; ICANS, immune effector cell‐associated neurotoxicity syndrome.

^a^ Tested at month 1/3 time point.

**Figure 2:**
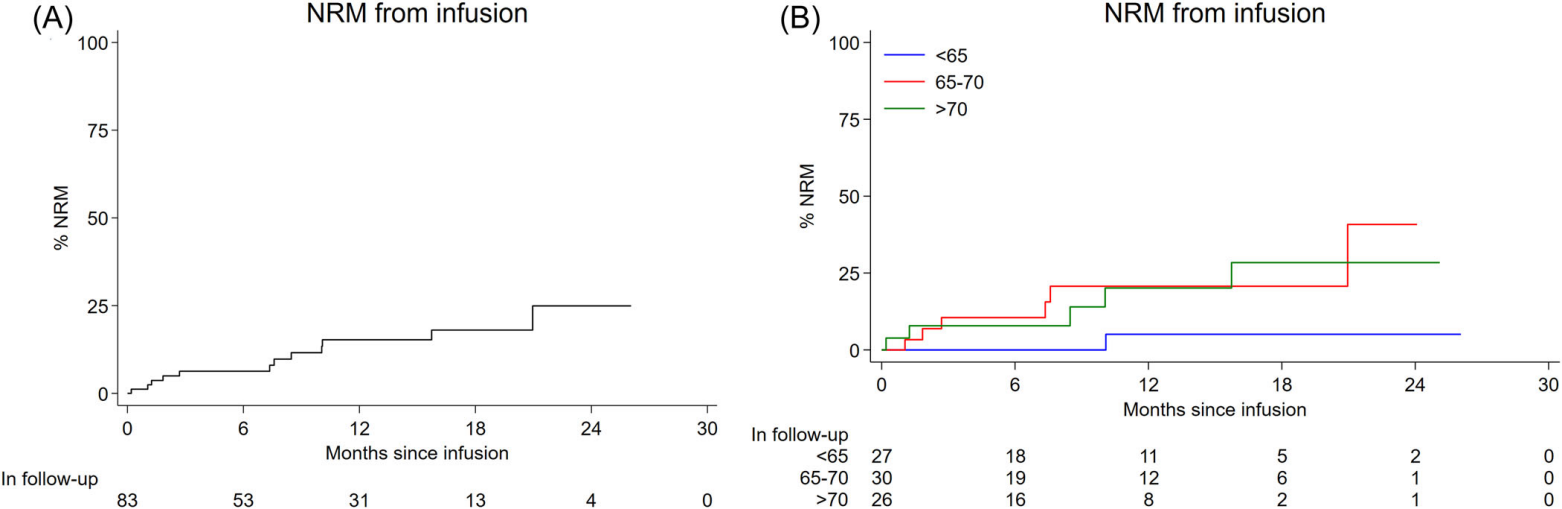
(A, B) Cumulative incidence of non‐relapse mortality from infusion for all patients (A) and by age (B). NRM, non‐relapse mortality.

At a median follow‐up of 13.3 months from infusion (IQR: 6.3–18.6), 12 NRM events were reported. The 24‐month cumulative incidence of NRM was 25% (95% CI: 13–45) with no reported events beyond this point (Figure 2A). On UVA, older patients were significantly more likely to experience an event (SHR = 1.12, 95% CI: 1.04–1.22, *p* = 0.01) (Figure 2B and Table S7); 11/12 events (92%) occurring in those ≥65 years. Male gender, LDH > ULN at submission and severe/life‐threatening infection within the first month were also significant. The majority of NRM events occurred beyond 90 days post‐infusion (58%, 7/12) and were mostly attributable to infection. SARS‐CoV2 accounted for 43% (3/7) of late deaths. Of late NRM events, 3/7 (43%) had a history of high‐grade CRS or ICANS during the initial inpatient stay. Of deaths within 90 days (*n* = 5), none of the patients had neutrophil recovery at time of death; bacterial sepsis accounting for 60% (3/5) (Table S8).

### Response and survival outcomes

**Figure 3:**
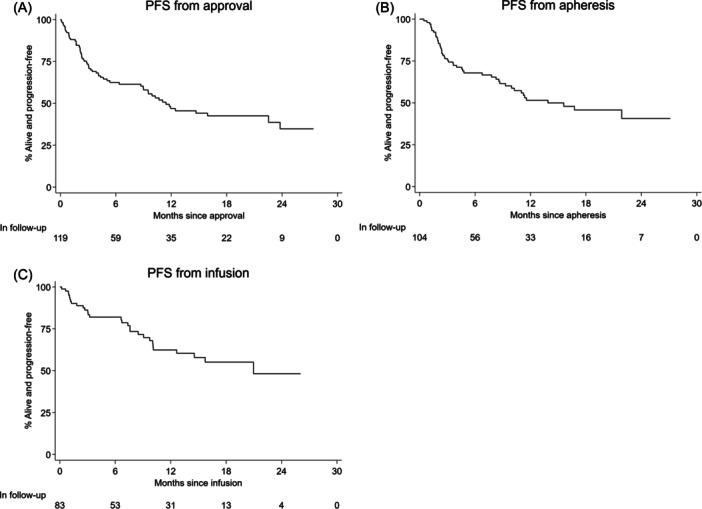
(A–C) Progression‐free survival (PFS) from approval (A), apheresis (B), and infusion (C) by Kaplan–Meier analysis.

**Figure 4:**
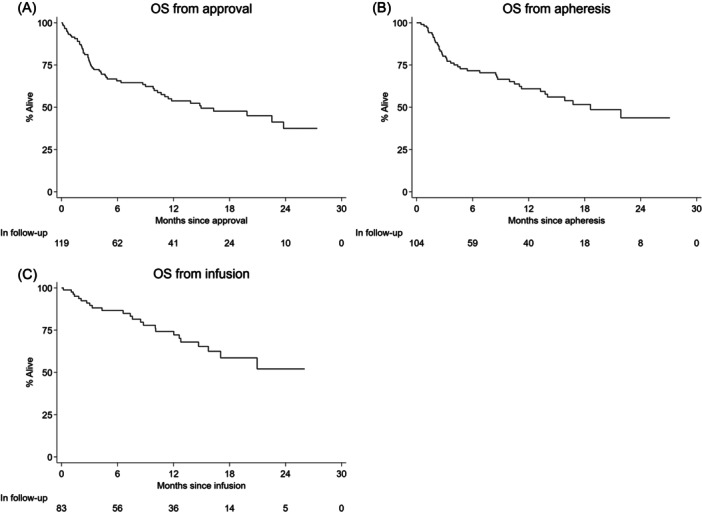
(A–C) Overall survival (OS) from approval (A), apheresis (B), and infusion (C) by Kaplan–Meier analysis.

Eighty‐three patients were evaluable for response; best ORR was 87% (81% CR and 6% PR). PFS and OS from approval, harvest, and infusion are demonstrated in Table S9, Figures 3A–C and 4A–C. Median PFS for all infused patients was 21 months (10.1–NA) with a 6‐ and 12‐month PFS of 82% (71–89) and 62% (49–73), respectively. Median OS was not reached for infused patients with 6‐ and 12‐month OS of 87% (76–93) and 74% (62–83), respectively (Table S9 and Figure 4C). Factors at submission associated with an inferior PFS included male sex, bulky disease >5 cm, and POD24 (Table S7). On MVA, bulky disease and male sex retained significance for both PFS and OS (Table S10). Factors pre‐LD associated with an inferior PFS included ≥3 EN sites and ECOG PS > 1 (Table S7). On MVA of pre‐LD variables, PFS and OS were independently associated with male sex, ECOG PS > 1 and a previous MF (Table S11). Post‐infusion, the occurrence of any grade CRS or ICANS had a protective survival benefit (Table S7).

**Figure 5:**
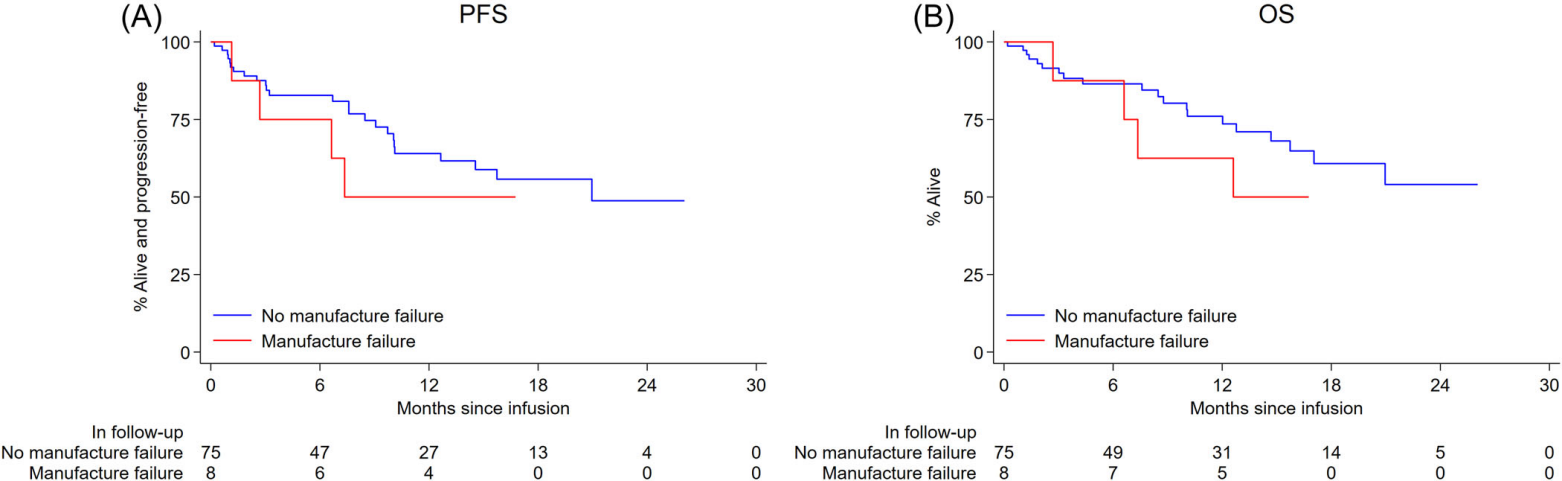
(A, B) Progression‐free survival outcome (A) and overall survival outcome (B) for infused patients with/without manufacturing failure.

**Figure 6:**
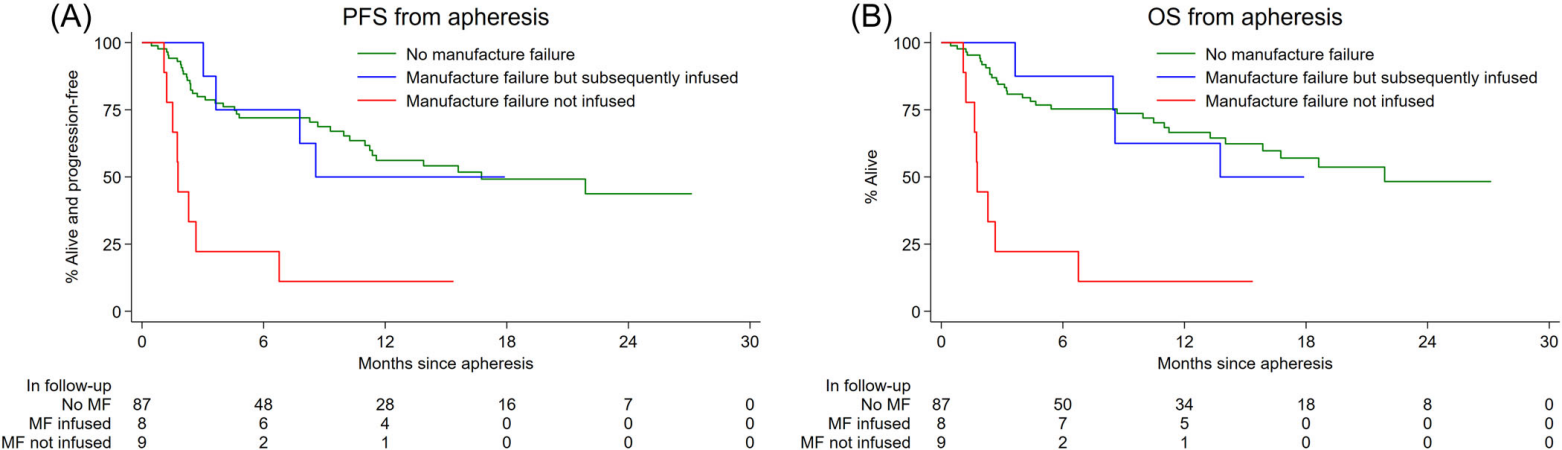
(A, B) Progression‐free survival outcome (A) and overall survival outcome (B) for all patients harvested with/without manufacturing failure. Note that the groups should not be directly compared as they are defined by events that occur at different points after the date of apheresis. For example, patients who had manufacturing failure and subsequent infusion must have had stable disease for long enough to undergo a second apheresis and receive the infusion to be counted in that group.

Eight patients were infused after a 1st MF and a subsequent successful second T‐cell harvest. Early relapses were noted in this group with a median PFS of 7.4 months (95% CI: 1.1–NA) relative to a median PFS of 21 months (10.1–NA) in non‐MF cohort (Figure 5A,B and Table S12). However, 50% of those with a previous MF remained in remission at month 12 suggesting that some can still derive benefit from this therapy. Survival outcomes for those with MF who did not reach cell infusion were dismal with a median OS of 1.8 months from apheresis (95% CI: 1.1–6.8) (Figure 6A,B).

## DISCUSSION

Brexu‐cel offers the potential for prolonged remissions in a subset of patients with r/r MCL after failure of BTKi, a particularly poor prognostic group. However, real‐world application of CAR T at 3rd line faces inherent challenges. Considering ITT, 30% of eligible and 20% of harvested patients did not receive their CAR T‐cell infusion, with PD the primary cause of drop‐out. Historically, a substantial proportion of MCL patients did not go on to receive further therapies after BTKi failure; 57% and 37.9% in the United Kingdom^9^ and SCHOLAR‐2^11^ datasets respectively, with a dismal median OS of 0.4 months. Relative to diagnostic disease risk profiles, a predominance of blastoid phenotype, bulky disease, Ki‐67 > 30%/ > 50% and ECOG ≥ 2 have been demonstrated post BTKi failure.^11^ This mirrors many of the disease features noted among those unfit for further therapy thereafter.^9^ Where data were available, 38%, 38%, and 76% of our patients had blastoid/pleomorphic disease, a *TP53* mutation and Ki‐67 ≥ 30% at submission, respectively. The frequent use of pre‐apheresis BT, including chemoimmunotherapy, to stabilize disease after approval also highlights the burden and kinetics of disease at relapse. Taken together, we can hypothesize that MCL patients relapsing on a BTKi in the real‐world are challenged by high‐risk disease features, rapid disease kinetics, and/or frailty.

Capturing patients failing 2nd line BTKi prior to florid relapse may improve the feasibility and outcome of CAR T treatment, particularly in high‐risk candidates. Bulky disease (>5 cm) at submission was independently associated with inferior PFS and OS post‐infusion in our cohort. Disease burden variables at submission such as higher LDH and circulating disease were also associated with a higher risk of drop out and MF, respectively. Repeat biopsies at the point of submission for CAR T were not performed in all cases. MCL subtype and *TP53* mutation status were unknown at submission in 34% and 53% of patients, respectively and the risk of drop out associated with specific high‐risk features therefore cannot be estimated. With a view to improving outcomes, British Society of Haematology (BSH) guidance proposes a risk‐based surveillance strategy, for potential CAR T candidates commencing ibrutinib at 2nd line.^19^ Predictors for shorter PFS on ibrutinib such as disease bulk ≥5 cm, high‐risk sMIPI score, POD24, *TP53* mutations, and blastoid histology are considered high‐risk,^24^, ^25^, ^26^ warranting an early response assessment on BTKi and referral to a CAR T centre at the first sign of ibrutinib failure. Of note, the marketing authorization for brexu‐cel differs between the EMA and FDA, where exposure to a BTKi is not a requirement for the latter. 2nd line CAR T trials should also be explored.

The use of post‐apheresis BT is associated with high‐risk disease features in large B‐cell lymphoma (LBCL)^27^, ^28^ but has the potential to stabilize disease and maintain performance status prior to infusion, without compromising outcome.^27^ Achieving a CR or PR to BT in LBCL has been shown to confer an independent survival benefit, reducing the risk of death or progression post CAR T by 42%.^29^ BT practice is also supported by the correlation between tumor burden pre‐infusion and the risk of higher‐grade toxicity^30^ and early relapse.^18^, ^31^ In ZUMA‐2, BT was limited to a BTKi and/or steroids and administered to 37% of patients. Outside of clinical trials, BT is administered to 68%–82% of MCL patients.^15^, ^16^, ^17^ Ninety percent of our patients received post harvest therapy achieving an ORR of 41%, with 19% of patients receiving >1 modality of BT. No association between BT response and outcome was noted. However, akin to LBCL, variables such as bulk, EN sites, and ECOG PS > 1 were associated with inferior survival outcomes, inferring the potential for improvement with effective BT strategies. Agents such as pirtobrutinib, venetoclax, and bispecific antibodies are not readily available in the United Kingdom outside of clinical trials. Pirtobrutinib, as a single agent, has demonstrated an ORR of 57.8% in those previously exposed to a covalent BTKi^32^ and may be a promising and well‐tolerated BT option.

At 16.3%, the rate of MF in our cohort was significantly higher than in ZUMA‐2 (4%),^12^ United States^16^ (3.7%), and European^15^ (8%) real‐world data sets. The definition of MF can vary, often including out of specification (OOS) products. In our cohort, MF was defined as failure of the leukapheresis material to successfully yield a CAR T‐cell product that could be requested by the CAR T physician and was considered separately to OOS. There was no association between MF and CD3^+^ cell count in peripheral blood, total CD3^+^ cells harvested, number of lines of therapy, or Bendamustine exposure/timing. On UVA, MF was associated with features of disease burden, including LDH > ULN and circulating disease in the peripheral blood on the day of harvest. In the absence of detailed quantitative and qualitative analysis of the harvested material,^33^ these findings are potentially suggestive of a disease‐associated qualitative T‐cell defect hampering cell expansion during manufacture. Wang et al. have previously demonstrated that T‐cells in the MCL tumor microenvironment (TME) demonstrate decreased expression of activation markers with parallel increased expression of markers associated with exhaustion and senescence,^34^ a well‐recognized phenomenon in cancer.^35^ Although, the manufacture of brexu‐cel encompasses a T‐cell selection step to remove contaminating cells from the harvest material, it is conceivable that circulating MCL cells in the peripheral blood, namely the TME, may contribute to inherent T‐cell dysfunction and failure of expansion during manufacture in such patients. Other groups have demonstrated that MF rates are higher in those who have received Bendamustine.^16^, ^36^, ^37^ Only one patient with MF had received Bendamustine within 6 months of harvest. Acknowledging small numbers, no association between Bendamustine and MF was found. Outcomes for patients with B‐cell malignancies receiving OOS CAR T products appear comparable to those with compliant products.^36^, ^38^, ^39^ However, on MVA in our cohort, PFS outcomes for those with ≥1 MF, who were subsequently infused after a second successful manufacture (*n* = 8), were inferior with a shorter median PFS of 7.4 months (95% CI: 1.1–NA) relative to a median PFS of 21 months (10.1–NA) in the non‐MF cohort. This may be indicative of poor persistence and T‐cell functionality in this subset. Although 50% remained in remission at 1 year post‐infusion, consolidative therapies could be considered in appropriate high‐risk candidates. Longer follow‐up and larger numbers are needed to assess durability of response and the contributing factors in such patients. The lower incidence of MF in 2022/23 may be related to an initial backlog of high‐burden patients in the first year of drug approval and/or emerging early data on the potential importance of disease control at cell harvest.^40^

Survival outcomes for patients infused with brexu‐cel in the United Kingdom are comparable to those achieved in ZUMA‐2^12^ and larger real‐world datasets.^15^, ^16^ Relatively poorer survival outcomes have been reported by other groups,^41^, ^42^ although short follow‐up and small numbers preclude firm conclusions. In our cohort, neither the use of Bendamustine prior to harvest nor the presence of high‐risk disease features such as *TP53* mutation, blastoid disease, high‐risk sMIPI score or Ki‐67 ≥ 50% were associated with an inferior PFS or OS. On MVA, bulky disease at submission, male sex, ECOG PS > 1 pre‐LD, and MF had a negative impact on survival. Of note, our survival outcomes diverge from ZUMA‐2 and other real‐world reports when we consider all patients initially proposed for brexu‐cel; our ITT cohort had a median PFS of 11.4 months (Table S9), highlighting the challenges of real‐world delivery. This may be reflective of how trial logistics with strict eligibility criteria can exclude the most representative sample of candidates. Consideration of ITT outcome is important as we counsel our patients and assess the risk: benefit of brexu‐cel compared to newer targeted treatments competing for the 3rd line MCL space. With the advent of CAR T therapy for MCL, allograft has fallen out of favor. ASTCT, CIBMTR, and EBMT guidance currently recommends consideration of CAR T over allograft, where CAR T is available^43^ (Grade C Recommendation). A higher rate of drop out in younger patients in our cohort conflicts somewhat with data in LBCL, where ASCT‐unfit patients, a predominantly older cohort, were less likely to reach cell infusion.^44^ It raises questions on the role of allograft in select younger fit candidates with high‐risk disease in first remission or responding to a covalent BTKi. In the absence of more effective bridging strategies, rapid refractory PD post‐BTKi failure is a concern, and feasibility of CAR T treatment at 3rd line cannot be guaranteed.

Although rates of high‐grade CRS, ICANS, and infection were in‐keeping with the expected toxicity profile of brexu‐cel, extended follow‐up of our cohort demonstrated a cumulative incidence of NRM of 6%, 15%, and 25% at 6 months, 1, and 2 years, respectively. The HEMATOTOX (HT) score has been shown to delineate those at higher risk of NRM, severe infections, and haematotoxicity post‐brexu‐cel.^17^ In our analysis, risk factors for NRM included older age, male sex, LDH > ULN, and severe or life‐threatening infection with 1 month of infusion. The excess of late NRM events (>90 days, 58%) is of concern, with SARS‐CoV2 accounting for 43% (3/7). Despite the reduced incidence of SARS‐CoV2‐related mortality in recent years (likely attributable to the combination of less virulent variants, vaccination programs, and pre‐emptive pharmacotherapies), this group remain vulnerable.^45^ Vaccine‐induced T‐cell responses may provide some protection to those with B‐cell aplasia and impaired antibody production.^46^ Older patients are at higher risk of NRM, despite a preserved ECOG PS pre‐LD (Tables S7 and S8), suggesting that more sensitive measures of “fitness” may be required. Distinct from age and ECOG PS, frailty is defined as a state of increased vulnerability or lower resilience in response to acute stressors and is associated with adverse outcomes.^47^ There are limited data on frailty and the rehabilitation needs of older CAR T patients (pre‐infusion and post‐infusion) but there is increasing awareness of this clinical need.^48^ Given the median age at diagnosis of MCL is 68 years and the majority of those failing ≥2 lines of therapy will be >70 years, rigorous patient selection is a key component of harnessing the benefits of this CAR T product. Close observation for (and investigation of) late haematotoxicity, including early consideration of a stem cell boost where appropriate, adherence to prophylaxis guidance, use of intravenous immunoglobulin, and post‐CAR T vaccination are important components in maintaining longer‐term safety post‐infusion.^49^, ^50^ Lisocabtagene maraleucel (Liso‐cel), recently licensed by the FDA, has demonstrated durable responses with a more favorable toxicity profile in a similar cohort of patients^51^ and may represent an alternative option in less robust candidates.

Our ITT analysis is limited by retrospective data collection (despite prospective patient approval), somewhat limited repeat biopsies at submission, lack of central response assessment, and of pharmacokinetic data. Eligibility for ZUMA‐2 was not assessed. Data on the impact of manufacturing/harvest slot availability and physician decision to give apheresis BT were not recorded. Our analysis also assumes that physicians adhered to recommended drug washout periods prior to T‐cell harvest. Although exact cell delivery dates were not recorded, median time from harvest to infusion in our cohort was 36 days. UK turnaround times are therefore longer than ZUMA‐2^12^ (median of 16 days to cell delivery) but comparable to United States^16^ (median of 28 days to LD) and European^15^ (median of 41 days to infusion) real‐world data sets.

In summary, brexu‐cel can achieve high response rates and durable remissions in a subset of MCL patients after ≥2 lines of therapy. ITT analysis highlights that a significant proportion of eligible patients fail to reach cell infusion due to PD or MF. Detection of early failure of ibrutinib and more effective bridging strategies will be key factors in reducing drop out and improving survival outcomes. NRM events, early and late, warrant further attention.

## AUTHOR CONTRIBUTIONS

Maeve A. O'Reilly and William Wilson led on the design of this document and prepared the manuscript. William Wilson also analysed the data. David Burns, Andrea Kuhnl, Frances Seymour, Ben Uttenthal, Caroline Besley, Rajesh Alajangi, Thomas Creasey, Shankara Paneesha, Johnathon Elliot, Carlos Gonzalez Arias, Sunil Iyengar, Matthew R. Wilson, Alison Delaney, Lourdes Rubio, Jonathan Lambert, Khalil Begg, Stephen Boyle, Kathleen P. L. Cheok, Rod Johnson, Robin Sanderson contributed to data collection. Rod Johnson and Graham P. Collins lead the NCCP approval process. All authors contributed to the preparation and approval of the final submitted version.

## CONFLICT OF INTEREST STATEMENT

Maeve A. O'Reilly: honoraria from Kite Gilead, Novartis, and Janssen. Advisory boards Kite Gilead and Autolus. Conference/travel support Kite Gilead. William Wilson: No COI. David Burns: Kite Gilead consultancy fees and support for educational meetings. Andrea Kuhnl: Kite Gilead conference support, honoraria, advisory board, Novartis: honoraria, research funding, Advisory board: Roche, Abbvie, BMS. Frances Seymour: No COI. Ben Uttenthal: No COI. Caroline Besley: Honoraria: Kite, Janssen, Novartis and Takeda. Rajesh Alajangi: No COI. Thomas Creasey: No COI. Shankara Paneesha: No COI. Johnathon Elliot: No COI. Carlos Gonzalez Arias: Kite Gilead conference support, honoraria, advisory board, research funding; Novartis conference support, honoraria, advisory board; BMS advisory board. Sunil Iyengar: conference support Beigene, BMS, Takeda. Speaker fees Kite Gilead, Takeda. Advisory boards Kite, MSD. Matthew R. Wilson: Honoraria/speaker fees: Kite/Gilead, Janssen, Sobi, Takeda, Veriton Pharma, AstraZeneca, Roche. Conference/travel support: Gilead, Janssen, Kite, Takeda, AstraZeneca. Alison Delaney: Kite Gilead conference support. Lourdes Rubio: No COI. Jonathan Lambert: advisory boards: Kite‐Gilead and Blueprint Pharmaceuticals; BMS, Takeda and Novartis: conference support. Khalil Begg: No COI. Stephen Boyle: Honoraria Kite Gilead. Kathleen P. L. Cheok: No COI. Graham P. Collins: Kite Gilead speaker fees, advisory board. Claire Roddie: Advisory boards and speakers fees Novartis, Kite Gilead, BMS, Amgen, Autolus. Rod Johnson: Kite Gilead consultancy fees, support for educational meetings. Robin Sanderson: Kite Gilead speakers bureau, honoraria, conference travel, Novartis speakers bureau, honoraria, conference travel.

## FUNDING

The authors declare no sources of funding.

## Supporting information

Supporting Information.

## Data Availability

The data that support the findings of this study are available on request from the corresponding author. The data are not publicly available due to privacy or ethical restrictions. Data may be shared upon reasonable request via email to the corresponding author.
