# Supplementary material for: Brexucabtagene autoleucel for relapsed or refractory mantle cell lymphoma in the United Kingdom: A real‐world intention‐to‐treat analysis
Source: Hemasphere. 2024 Jun 13;8(6):e87. doi: 10.1002/hem3.87 (PMC11170269; doi:10.1002/hem3.87)
Supplement: Supplementary file 1 — Supporting Information. [file HEM3-8-e87-s001.docx]

**Table S1:**

| **CAR T centres** |
| --- |
| University College London Hospital, London |
| Kings College Hospital, London |
| University Hospital Birmingham |
| Leeds Teaching Hospital |
| Cambridge University Hospital |
| University Hospital Bristol |
| The Christie NHS foundation Trust, Manchester |
| Queen Elizabeth University Hospital, Glasgow |
| Newcastle upon Tyne Hospitals |
| Royal Marsden London |
| Sheffield Teaching Hospitals |
| Manchester Royal Infirrmary |

**Table S2: NCCP eligibility criteria for Tecartus**

| **NHS England NCCP eligibility criteria** | | **Comments** |
| --- | --- | --- |
| **Diagnosis** | MCL with t(11;14) or cyclin D1 overexpression |  |
| **Age** | No upper age limit | Suitability at discretion of CAR T-cell centre |
| **Previous Treatment** | - Anthracycline or bendamustine or high-dose cytarabine-containing regimen   *and*   - Anti-CD20 monoclonal antibody   *and*   - BTKi (ibrutinib/acalabrutinib/other BTKi) | Prior allograft is not an exclusion |
| **Patient** | - ECOG PS 0-1 at assessment (ECOG PS 2 at infusion is acceptable) - No active CNS disease - HIV/Hepatitis B/Hepatitis C negative or undetectable viral load | A prior history of MCL in the CNS is not an exclusion  Medical co-morbidities at discretion of CAR T-cell centre |

NCCP, National CAR T Clinical Panel; NHS, National Health Service; MCL, mantle cell lymphoma; BTKi, Bruton tyrosine kinase inhibitor; ECOG PS, Eastern Cooperative Oncology Group Performance Status; CNS, central nervous system; HIV, human immunodeficiency virus

**Table S3: Univariate analysis of factors associated with failure to reach cell harvest and/or cell infusion ^a^**

|  | **Characteristic at submission/approval ^b^** | **N failed to infuse due to PD ^d^/Total N** | **OR ^c^ (95% CI), p-value** |
| --- | --- | --- | --- |
| **Continuous variables** | |  |  |
| Age | | 18/100 (18%) | 0.94 (0.89-1.00), p=**0.05** |
| LDH | | 17/98 (17%) | 1.14 (1.00-1.30), p=**0.05** |
| CD3 count in peripheral blood (1^st^ apheresis) | | 8/64 (13%) | 1.44 (0.78-2.69), p=0.25 |
| Total CD3+ cells harvested (1^st^ apheresis) | | 6/56 (11%) | 1.05 (0.99-1.11), p=0.12 |
| **Categorical variables** | |  |  |
| Age | |  |  |
| <65 | | 8/34 (24%) | Reference |
| 65-69 | | 6/32 (19%) | 0.75 (0.23-2.47), p=0.64 |
| ≥70 | | 4/34 (12%) | 0.43 (0.12-1.61), p=0.21 |
| Sex | |  |  |
| Female | | 4/26 (15%) | Reference |
| Male | | 14/74 (19%) | 1.28 (0.38-4.32), p=0.69 |
| Subtype | |  |  |
| Other | | 5/38 (13%) | Reference |
| Blastoid/pleomorphic | | 7/28 (25%) | 2.20 (0.62-7.84), p=0.22 |
| TP53 aberration | |  |  |
| No aberration | | 5/26 (19%) | Reference |
| Aberration | | 6/24 (25%) | 1.40 (0.37-5.37), p=0.62 |
| TP53 mutation | |  |  |
| No mutation | | 7/30 (23%) | Reference |
| Mutation | | 3/18 (17%) | 0.66 (0.15-2.95), p=0.58 |
| Ki-67 | |  |  |
| <30% | | 2/13 (15%) | Reference |
| ≥30% | | 8/42 (19%) | 1.29 (0.24-7.03), p=0.77 |
| Bulk (>5cm) | |  |  |
| ≤5cm | | 12/66 (18%) | Reference |
| >5cm | | 6/34 (18%) | 0.96 (0.33-2.84), p=0.95 |
| LDH>ULN | |  |  |
| ≤ULN | | 5/53 (9%) | Reference |
| >ULN | | 12/45 (27%) | 3.49 (1.12-10.84), p=**0.03** |
| Extra-nodal disease | |  |  |
| No | | 1/25 (4%) | Reference |
| Yes | | 17/75 (23%) | 7.03 (0.89-55.87), p=**0.07** |
| No. extra-nodal sites | |  |  |
| <3 | | 14/87 (16%) | Reference |
| ≥3 | | 4/13 (31%) | 2.32 (0.63-8.58), p=0.21 |
| No. prior lines | |  |  |
| 2 | | 12/61 (20%) | Reference |
| >2 | | 6/39 (15%) | 0.74 (0.25-2.17), p=0.59 |
| POD24 | |  |  |
| No | | 6/43 (14%) | Reference |
| Yes | | 12/56 (21%) | 1.68 (0.58-4.92), p=0.34 |
| Circulating disease (1^st^ apheresis) | |  |  |
| No | | 6/70 (9%) | Reference |
| Yes | | 4/20 (20%) | 2.67 (0.67-10.59), p=0.16 |
| Lymphocyte count in PB x10^9^/L (1^st^ apheresis) | |  |  |
| <5 | | 6/74 (8%) | Reference |
| >5 | | 3/17 (18%) | 2.43 (0.54-10.89), p=0.25 |
| Age | |  |  |
|  | <65 | 8/34 (24%) | Reference |
|  | 65-69 | 6/32 (19%) | 0.75 (0.23-2. 47), p=0.64 |
|  | ≥70 | 4/34 (12%) | 0.43 (0.12-1.61), p=0.21 |
| ECOG PS | |  |  |
|  | 0 | 3/35 (9%) | Reference |
|  | 1 | 15/65 (23%) | 3.20 (0.86-11.94), p=**0.08** |
| Stage | |  |  |
|  | I-II | 1/12 (8%) | Reference |
|  | III | 1/9 (11%) | 1.37 (0.07-25.43), p=0.83 |
|  | IV | 16/79 (20%) | 2.79 (0.34-23.26), p=0.34 |
| sMIPI | |  |  |
|  | Low | 3/18 (17%) | Reference |
|  | Intermediate | 6/29 (21%) | 1.30 (0.28-6.03), p=0.73 |
|  | High | 8/38 (21%) | 1.33 (0.31-5.77), p=0.70 |
| Closest bendamustine to 1^st^ apheresis | |  |  |
|  | None | 8/56 (14%) | Reference |
|  | <6 months | 1/11 (9%) | 0.60 (0.07-5.35), p=0.65 |
|  | 6-24 months | 2/11 (18%) | 1.33 (0.24-7.34), p=0.74 |
|  | >24 months | 1/15 (7%) | 0.43 (0.05-3.73),0.44 |

^a^ where progressive disease (PD) deemed to be the primary reason for drop out by treating physician. Patients with manufacturing failure were excluded.

^b^ LDH: lactate dehydrogenase; ULN: upper limit of normal; PB: peripheral blood; ECOG PS: Eastern Cooperative Oncology Group performance status; POD24; Progression of disease within 24 months of front-line therapy; HCT-CI: Hematopoietic Cell Transplantation-specific Comorbidity Index; sMIPI: simplified MCL international prognostic index

^c^ Fisher’s exact used when odds ratio could not be calculated

^d^ N=1 patient excluded from analysis as infused with CNS PD on compassionate access

**Table S4: Univariate analysis of factors associated with manufacturing failure**

|  | **Characteristic ^a^** | **N with manufacture failure ^b^/Total N** | **OR ^c^ (95% CI), p-value** |
| --- | --- | --- | --- |
| **Continuous variables** | |  |  |
| Age | | 16/103 (16%) | 1.02 (0.95-1.09), p=0.65 |
| LDH at submission | | 15/101 (15%) | 1.14 (0.96-1.36), p=0.13 |
| WCC in PB on day of harvest (x10^9^/L) | | 15/102 (15%) | 1.02 (1.00-1.05), p=**0.02** |
| Lymph count in PB on day of harvest (x10^9^/L) | | 16 (16%) | 1.03 (1.00-1.05), p=**0.05** |
| CD3 count in PB on day of harvest (x10^9^/L) | | 10 (14%) | 1.13 (0.59-2.18), p=0.71 |
| Total CD3+ cells harvested | | 12/64 (19%) | 0.86 (0.70-1.07), p=0.18 |
| **Categorical variables** | |  |  |
| Sex | |  |  |
| Female | | 5/28 (18%) | Reference |
| Male | | 11/75 (15%) | 0.79 (0.25-2.52), p=0.69 |
| Subtype | |  |  |
| Other | | 9/41 (22%) | Reference |
| Blastoid/pleomorphic | | 3/28 (11%) | 0.43 (0.10-1.74), p=0.24 |
| TP53 aberration | |  |  |
| No aberration | | 3/24 (13%) | Reference |
| Aberration | | 5/25 (20%) | 1.75 (0.37-8.30), p=0.48 |
| TP53 mutation | |  |  |
| No mutation | | 5/29 (17%) | Reference |
| Mutation | | 3/17 (18%) | 1.03 (0.21-4.97), p=0.97 |
| Ki-67 | |  |  |
| <30% | | 5/29 (17%) | Reference |
| ≥30% | | 3 (18%) | 1.01 (0.18-5.59), p=0.99 |
| Bulk | |  |  |
| ≤5cm | | 11/66 (17%) | Reference |
| >5cm | | 5/37 (14%) | 0.78 (0.25-2.45), p=0.67 |
| LDH | |  |  |
| ≤ULN | | 4/55 (7%) | Reference |
| >ULN | | 11/46 (24%) | 4.01 (1.18-13.61), p=**0.03** |
| Extra-nodal disease | |  |  |
| No | | 4/25 (16%) | Reference |
| Yes | | 12/78 (15%) | 0.95 (0.28-3.28), p=0.94 |
| No. extra-nodal sites | |  |  |
| <3 | | 15/91 (16%) | Reference |
| ≥3 | | 1/12 (8%) | 0.46 (0.06-3.84), p=0.47 |
| No. prior lines | |  |  |
| 2 | | 10/63 (16%) | Reference |
| >2 | | 6/40 (15%) | 0.94 (0.31-2.81), p=0.91 |
| Prior ASCT | | 6/15 |  |
| No | | 9/67 (13%) | Reference |
| Yes | | 6/34 (18%) | 1.38 (0.45-4.26), p=0.57 |
| Prior Allo-SCT | |  |  |
| No | | 13/88 (15%) | Reference |
| Yes | | 3/15 (20%) | 1.44 (0.36-5.82), p=0.61 |
| POD24 | |  |  |
| No | | 6/41 (15%) | Reference |
| Yes | | 10/61 (16%) | 1.14 (0.38-3.44), p=0.81 |
| Pre-apheresis bridging | |  |  |
| No | | 8/44 (18%) | Reference |
| Yes | | 8/59 (14%) | 0.71 (0.24-2.06), p=0.52 |
| Type pre-apheresis bridging (chemo vs other) | |  |  |
| Other | | 4/37 (11%) | Reference |
| Chemotherapy | | 4/22 (18%) | 1.83 (0.41-8.22), p=0.43 |
| Bendamustine before 1^st^ apheresis | |  |  |
| No | | 9/61 (15%) | Reference |
| Yes | | 7/42 (17%) | 1.16 (0.39-3.39), p=0.79 |
| Closest bendamustine to 1^st^ apheresis | | | |
|  | None | 9/61 (15%) | Reference |
|  | <6 months | 1/12 (8%) | 0.53 (0.06-4.58), p=0.56 |
|  | 6-24 months | 4/14 (29%) | 2.31 (0.59-8.99), p=0.23 |
|  | >24 months | 2/15 (13%) | 0.89 (0.17-4.62), p=0.89 |
| Circulating disease at 1^st^ apheresis | |  |  |
| No | | 9/76 (12%) | Reference |
| Yes | | 7/25 (28%) | 2.90 (0.95-8.84), p=**0.06** |
| Lymphocyte count in PB 1^st^ apheresis (x10^9^/L) | |  |  |
|  | <5 | 9/81 (1%) | Reference |
|  | 5-10 | 0/7 (0%) | p=1 |
|  | 10-30 | 3/9 (33%) | 4.00 (0.85-18.84), p=0.08 |
|  | >30 | 4/6 (67%) | 16.00 (2.56-100.08), p<**0.01** |
| ECOG PS | |  |  |
|  | 0 | 7/37 (19%) | Reference |
|  | 1 | 9/66 (14%) | 0.68 (0.23-2.00), p=0.48 |
| Stage | |  |  |
|  | I-II | 1/11 (9%) | Reference |
|  | III | 1/9 (11%) | 1.25 (0.07-23.26), p=0.88 |
|  | IV | 14/82 (17%) | 2.06 (0.24-17.40), p=0.51 |
| sMIPI | |  |  |
|  | Low | 2/20 (10%) | Reference |
|  | Intermediate | 4/28 (14%) | 1.50 (0.25-9.11), p=0.66 |
|  | High | 6/39 (15%) | 1.64 (0.30-8.96), p=0.57 |

^a^ Characteristics at time of 1^st^ harvest. LDH: lactate dehydrogenase; WCC: white cell count; PB: peripheral blood; ULN: upper limit of normal; ECOG PS: Eastern Cooperative Oncology Group performance status; sMIPI: simplified MCL international prognostic index; ASCT; autologous stem cell transplant; POD24; Progression of disease within 24 months of front-line therapy.

^b^ N=1 case excluded from analysis due to prolonged “hold time” prior to manufacture

^c^ Fisher’s exact used when odds ratio could not be calculated

**Table S5: Bridging Therapy administered to MCL patients before and after T-cell apheresis**

| **Bridging Therapy pre apheresis** | **N=66** |
| --- | --- |
| R-cytarabine | 14 |
| RCHOP | 6 |
| R-Benda/BAC | 6 |
| Chlorambucil | 1 |
| BTKi +/- steroids +/- Rituximab | 20 |
| Steroids +/- Rituximab | 12 |
| Venetoclax | 1 |
| Radiotherapy | 6 |
| **Bridging therapy post apheresis** | **N=94** |
| **Chemotherapy** | |
| R-BAC | 24 |
| R-Benda | 6 |
| RCHOP | 6 |
| R-Cytarabine | 6 |
| Other R-Chemo | 1 |
| R-chemo + targeted therapy^1^ | 6 |
| **BTKi + other targeted therapies** | |
| Ibrutinib +/- steroids | 12 |
| Ibrutinib + Venetoclax +/- Ritux | 5 |
| Acalabrutinib | 1 |
| Pirtobrutinib | 2 |
| Venetoclax | 4 |
| **Radiotherapy +/- other** | |
| RT alone | 8 |
| R-chemo^2^ + RT | 10 |
| Targeted therapy + RT | 2 |
| Steroids | 1 |

**^1^**Sequential

^2^n=7 R-Benda based

**Table S6:** **Univariate analysis of factors associated with ≥grade 3 CRS**

| **Variables ^a^** | | **N ≥grade 3 CRS/Total N** | **OR (95% CI), p-value** |
| --- | --- | --- | --- |
| **At submission** | |  |  |
| Age | | 10/83 (12%) | 0.98 (0.90-1.07), p=0.69 |
| Sex | |  |  |
| Female | | 2/23 (9%) | Reference |
| Male | | 8/60 (13%) | 1.62 (0.32-8.25), p=0.56 |
| Subtype | |  |  |
| Other | | 6/34 (18%) | Reference |
| Blastoid/pleomorphic | | 2/21 (10%) | 0.49 (0.09-2.70), p=0.41 |
| TP53 aberration | |  |  |
| No aberration | | 1/22 (5%) | Reference |
| Aberration | | 4/18 (22%) | 6.00 (0.61-59.44), p=0.13 |
| TP53 mutation | |  |  |
| No mutation | | 1/24 (4%) | Reference |
| Mutation | | 3/15 (20%) | 5.75 (0.54-61.41), p=0.15 |
| Ki-67 | |  |  |
| <30% | | 0/11 (0%) | Reference |
| ≥30% | | 5/35 (14%) | p=0.32 |
| Extra-nodal disease | |  |  |
| No | | 4/24 (17%) | Reference |
| Yes | | 6/59 (10%) | 0.57 (0.14-2.22), p=0.41 |
| No. extra-nodal sites | |  |  |
| <3 | | 6/74 (8%) | Reference |
| ≥3 | | 4/9 (44%) | 9.07 (1.91-43.03), p=**0.01** |
| POD24 | |  |  |
| No | | 1/37 (3%) | Reference |
| Yes | | 9/45 (20%) | 9.00 (1.08-74.76), p=**0.04** |
| **Pre-LD** | |  |  |
| Manufacturing failure | |  |  |
| No | | 10/75 (13%) | Reference |
| Yes | | 0/8 (0%) | p=0.59 |
| Bulk pre-LD | |  |  |
| ≤5cm | | 5/57 (9%) | Reference |
| >5cm | | 5/19 (26%) | 3.71 (0.94-14.66), p=0.06 |
| Bulk pre-LD | |  |  |
| ≤10cm | | 6/57 (11%) | Reference |
| >10cm | | 3/7 (43%) | 6.38 (1.14-35.58), p=**0.03** |
| Extra-nodal disease | |  |  |
| No | | 4/24 (17%) | Reference |
| Yes | | 6/59 (10%) | 0.57 (0.14-2.22), p=0.41 |
| No. extra-nodal sites | |  |  |
| <3 | | 6/74 (8%) | Reference |
| ≥3 | | 4/9 (44%) | 9.07 (1.91-43.03), **p=0.01** |
| LDH pre-LD | |  |  |
| ≤ULN | | 7/45 (16%) | Reference |
| >ULN | | 3/30 (10%) | 0.60 (0.14-2.54), p=0.49 |
| ECOG PS pre LD | |  |  |
| 0 |  | 3/30 (10%) | Reference |
| 1 | | 4/47 (9%) | 0.84 (0.17-4.03), p=0.82 |
| 2 | | 3/5 (60%) | 13.50 (1.57-115.94), p=**0.02** |

^a^ CRS: cytokine release syndrome, POD24: progression of disease within 24 months of front-line therapy: LD: lymphodepletion; LDH: lactate dehydrogenase; ECOG PS: Eastern Cooperative Oncology Group performance status

**Figure S1: Severe/life-threatening infections within 1 month of infusion**


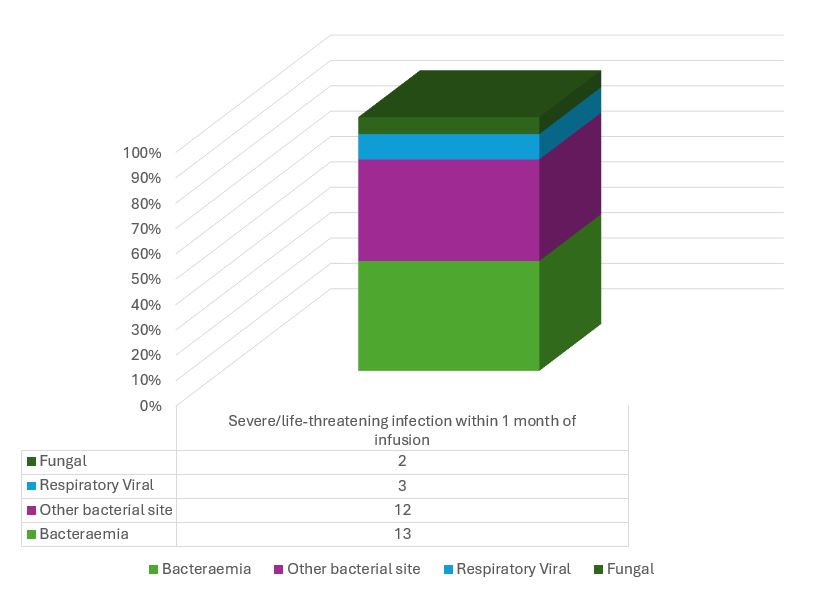


**Table S7: Univariate analysis of PFS, OS and NRM from infusion**

|  | **Variables ^a^** | **N** | | **PFS** | **OS** | **NRM ^b^** |
| --- | --- | --- | --- | --- | --- | --- |
| **At submission** | |  | |  |  |  |
| Age (continuous) | | 83 | | 1.04 (0.98-1.09), p=0.18 | 1.07 (1.01-1.14), p=**0.03** | 1.12 (1.04-1.22), p=**0.01** |
| Age | |  | |  |  |  |
| <65 | | 27/83 (33%) | | Reference | Reference | Reference |
| ≥65 | | 56/83 (67%) | | 1.25 (0.57-2.75), p=0.58 | 1.44 (0.60-3.48), p=0.42 | 6.77 (0.91-50.58), p=**0.06** |
| Sex | |  | |  |  |  |
| Female | | 23/83 (28%) | | Reference | Reference | Reference |
| Male | | 60/83 (72%) | | 7.13 (1.69-30.05), p=**0.01** | 5.52 (1.30-23.5), p=**0.02** | p=**0.02** |
| Subtype | |  | |  |  |  |
| Other | | 34/55 (62%) | | Reference | Reference | Reference |
| Blastoid/pleomorphic | | 21/55 (38%) | | 0.83 (0.32-2.14), p=0.70 | 1.35 (0.49-3.75), p=0.56 | 0.39 (0.05-3.24), p=0.38 |
| TP53 aberration | |  | |  |  |  |
| No aberration | | 22/40 (55%) | | Reference | Reference | Reference |
| Aberration | | 18/40 (45%) | | 1.26 (0.45-3.48), p=0.66 | 0.76 (0.22-2.61), p=0.67 | p=0.05 |
| TP53 mutation | |  | |  |  |  |
| No mutation | | 24/39 (62%) | | Reference | Reference | Reference |
| Mutation | | 15/39 (38%) | | 1.30 (0.46-3.68), p=0.62 | 1.11 (0.32-3.84), p=0.86 | p=0.09 |
| Ki67 | |  | |  |  |  |
| <30% | | 11/46 (24%) | | Reference | Reference | Reference |
| ≥30% | | 35/46 (76%) | | 1.36 (0.38-4.86), p=0.64 | 1.02 (0.28-3.75), p=0.98 | 0.63 (0.12-3.22), p=0.57 |
| LDH | |  | |  |  |  |
| ≤ULN | | 48/82 (59%) | | Reference | Reference | Reference |
| >ULN | | 34/82 (41%) | | 1.99 (0.94-4.25), p=0.07 | 1.96 (0.85-4.50), p=0.11 | 4.62 (1.35-15.82), p=**0.01** |
| Bulk (>5cm) | |  | |  |  |  |
| ≤5cm | | 54/83 (65%) | | Reference | Reference | Reference |
| >5cm | | 29/83 (35%) | | 2.68 (1.28-5.62), p=**0.01** | 2.70 (1.20-6.10), p=**0.02** | 2.28 (0.75-6.93), p=0.15 |
| No. extra-nodal sites | |  | |  |  |  |
| <3 | | 74/83 (89%) | | Reference | Reference | Reference |
| ≥3 | | 9/83 (11%) | | 1.46 (0.55-3.85), p=0.44 | 0.98 (0.29-3.31), p=0.98 | 0.57 (0.06-5.18), p=0.61 |
| ECOG | |  | |  |  |  |
| 0 | | 33/83 (40%) | | Reference | Reference | Reference |
| 1 | | 50/83 (60%) | | 1.56 (0.73-3.32), p=0.25 | 1.35 (0.60-3.06), p=0.47 | 1.17 (0.37-3.73), p=0.79 |
| Stage | |  | |  |  |  |
| I-II | | 11/83 (13%) | | Reference | Reference | Reference |
| III | | 8/83 (10%) | | 1.65 (0.27-9.96), p=0.58 | 1.53 (0.25-9.25), p=0.64 | 3.71 (0.39-35.16), p=0.25 |
| IV | | 64/83 (77%) | | 1.44 (0.34-6.12), p=0.62 | 0.94 (0.22-4.10), p=0.94 | 0.89 (0.10-7.57), p=0.92 |
| sMIPI | |  | |  |  |  |
| Low | | 15/69 (22%) | | Reference | Reference | Reference |
| Intermediate | | 23/69 (33%) | | 1.88 (0.50-7.11), p=0.35 | 2.57 (0.53-12.5), p=0.24 | 3.71 (0.39-35.16), p=0.25 |
| High | | 31/69 (45%) | | 1.90 (0.53-6.83), p=0.32 | 2.31 (0.49-10.9), p=0.29 | 0.89 (0.10-7.57), p=0.92 |
| No. prior lines | |  | |  |  |  |
| 2 | | 49/83 (59%) | | Reference | Reference | Reference |
| >2 | | 34 (41%) | | 1.22 (0.59-2.52), p=0.60 | 0.98 (0.44-2.20), p=0.97 | 1.11 (0.37-3.31), p=0.85 |
| Previous allograft | |  | |  |  |  |
| No | | 69 (83%) | | Reference | Reference | Reference |
| Yes | | 14/83 (17%) | | 1.01 (0.39-2.66), p=0.98 | 0.90 (0.31-2.63), p=0.84 | 0.41 (0.05-3.19), p=0.39 |
| POD24 | |  | |  |  |  |
| No | | 37 (45%) | | Reference | Reference | Reference |
| Yes | | 45/82 (55%) | | 2.54 (1.11-5.77), p=**0.03** | 2.18 (0.90-5.31), p=0.09 | 1.46 (0.42-5.07), p=0.55 |
| **At T-cell harvest** | |  | |  |  |  |
| Pre-apheresis bridging | |  | |  |  |  |
| No | | 34/83 (41%) | | Reference | Reference | Reference |
| Yes | | 49/83 (59%) | | 1.20 (0.57-2.54), p=0.63 | 0.90 (0.40-2.02), p=0.80 | 0.82 (0.27-2.45), p=0.72 |
| Type apheresis bridging | |  | |  |  |  |
| Other | | 32/49 (65%) | | Reference | Reference | Reference |
| Chemotherapy | | 17/49 (35%) | | 1.08 (0.40-2.92), p=0.88 | 1.38 (0.44-4.35), p=0.58 | 0.99 (0.18-5.42), p=0.99 |
| CD3+ count in PB (continuous) | | 56 | | 0.83 (0.47-1.48), p=0.54 | 0.74 (0.35-1.57), p=0.43 | 0.74 (0.42-1.31), p=0.30 |
| Total CD3+ cells harvested (continuous) | | 49 | | 0.95 (0.85-1.07), p=0.39 | 0.86 (0.69-1.07), p=0.18 | 0.84 (0.67-1.05), p=0.12 |
| Lymphocyte count in PB on day of harvest (x10^9^/L) | | | |  |  |  |
| <5 | | 70/83 (84%) | | Reference | Reference | Reference |
| 5-10 | | 4/83 (5%) | | 0.76 (0.10-5.62), p=0.79 | 0.93 (0.13-6.99), p=0.95 | 2.28 (0.24-21.76), p=0.47 |
| 10-30 | | 7/83 (8%) | | 0.87 (0.21-3.68), p=0.85 | 1.12 (0.26-4.79), p=0.88 | 1.01 (0.15-7.05), p=0.99 |
| >30 | | 2/83 (2%) | | 99.56 (8.73-1135.27), p<**0.01** | 7.49 (0.92-60.7), p=0.06 | p=0.6 |
| Circulating disease at first harvest | |  | |  |  |  |
| No | | 64/81 (79%) | | Reference | Reference | Reference |
| Yes | | 17/81 (21%) | | 2.03 (0.92-4.47), p=0.08 | 1.39 (0.55-3.51), p=0.48 | 1.95 (0.59-6.45), p=0.28 |
| Bendamustine before first apheresis | |  | |  |  |  |
| No | | 49/83 (59%) | | Reference | Reference | Reference |
| Yes | | 34/83 (41%) | | 1.52 (0.73-3.16), p=0.26 | 1.55 (0.69-3.46), p=0.29 | 1.28 (0.42-3.93), p=0.67 |
| Closest bendamustine to first apheresis | | |  |  |  |  |
| None | | 49/82 (60%) | | Reference | Reference | Reference |
| <6 months | | 10/82 (12%) | | 1.19 (0.39-3.64), p=0.76 | 0.95 (0.26-3.46), p=0.94 | 0.58 (0.70-4.84), p=0.62 |
| 6-24 months | | 9/82 (11%) | | 2.01 (0.72-5.61), p=0.18 | 2.47 (0.85-7.15), p=0.10 | 2.65 (0.74-9.46), p=0.13 |
| >24 months | | 14/82 (17%) | | 1.32 (0.47-3.67), p=0.60 | 1.30 (0.41-4.09), p=0.65 | 1.19 (0.23-6.09), p=0.83 |
| **Variables pre-LD** | |  | |  |  |  |
| LDH | |  | |  |  |  |
| ≤ULN | | 45/75 (60%) | | Reference | Reference | Reference |
| >ULN | | 30/75 (40%) | | 1.78 (0.85-3.75), p=0.13 | 2.50 (1.09-5.75), p=**0.03** | 1.63 (0.54-4.91), p=0.39 |
| Bulk | |  | |  |  |  |
| ≤5cm | | 57/76 (75%) | | Reference | Reference | Reference |
| >5cm | | 19/76 (25%) | | 1.89 (0.89-4.05), p=0.10 | 1.90 (0.82-4.41), p=0.13 | 2.27 (0.70-7.34), p=0.17 |
| No. extra-nodal sites | |  | |  |  |  |
| <3 | | 66/76 (87%) | | Reference | Reference | Reference |
| ≥3 | | 10/76 (13%) | | 2.75 (1.16-6.53), p=**0.02** | 2.65 (1.03-6.82), p=**0.04** | 0.60 (0.07-5.19), p=0.65 |
| Response to bridging | |  | |  |  |  |
| No response | | 40/75 (53%) | | Reference | Reference | Reference |
| Response (CR/PR) | | 35/75 (47%) | | 1.44 (0.66-3.13), p=0.36 | 1.29 (0.55-3.05), p=0.56 | 0.97 (0.30-3.16), p=0.96 |
| Manufacturing failure | |  | |  |  |  |
| No | | 75/83 (90%) | | Reference | Reference | Reference |
| Yes | | 8/83 (10%) | | 1.45 (0.50-4.19), p=0.49 | 1.78 (0.60-5.26), p=0.30 | 1.76 (0.38-8.10), p=0.47 |
| ECOG PS | |  | |  |  |  |
| 0 | | 30/82 (37%) | | Reference | Reference | Reference |
| 1 | | 47/82 (57%) | | 1.87 (0.81-4.32), p=0.14 | 2.09 (0.82-5.36), p=0.12 | 1.29 (0.39-4.23), p=0.68 |
| 2 | | 5/82 (6%) | | 6.00 (1.57-22.89), p=**0.01** | 4.40 (0.88-22.1), p=0.07 | 2.02 (0.19-21.01), p=0.56 |
| **Post infusion** | |  | |  |  |  |
| CRS any grade | |  | |  |  |  |
| No | | 6/83 (7%) | | Reference | Reference | Reference |
| Yes | | 77/83 (93%) | | 0.19 (0.07-0.58), **p<0.01** | 0.14 (0.04-0.45), **p<0.01** | 0.73 (0.11-4.74), p=0.74 |
| CRS grade 3+ | |  | |  |  |  |
| No | | 73/83 (88%) | | Reference | Reference | Reference |
| Yes | | 10/83 (12%) | | 1.41 (0.53-3.72), p=0.49 | 1.00 (0.30-3.37), p>0.99 | 1.30 (0.31-5.43), p=0.72 |
| ICANS any grade | |  | |  |  |  |
| No | | 37/83 (45%) | | Reference | Reference | Reference |
| Yes | | 46/83 (55%) | | 0.46 (0.22-0.97**), p=0.04** | 0.48 (0.21-1.09), p=0.08 | 1.65 (0.51-5.36), p=0.40 |
| ICANS grade 3+ | |  | |  |  |  |
| No | | 64/83 (77%) | | Reference | Reference | Reference |
| Yes | | 19/83 (23%) | | 0.49 (0.17-1.43), p=0.19 | 0.64 (0.22-1.87), p=0.41 | 1.87 (0.57-6.15), p=0.30 |
| Severe/life-threatening infection within 30 days ^c^ | | | |  |  |  |
| No | | 59/83 (71%) | | Reference | Reference | Reference |
| Yes | | 24/83 (29%) | | 1.59 (0.67-3.81), p=0.29 | 1.72 (0.72-4.12), p=0.23 | 4.63 (1.30-16.55), **p=0.02** |

^a^ PFS: progression-free survival; OS: overall survival; NRM: non-relapse mortality; LDH: lactate dehydrogenase; ULN: upper limit of normal; ECOG PS: Eastern Cooperative Oncology Group performance status; sMIPI: simplified MCL international prognostic index; POD24; Progression of disease within 24 months of front-line therapy**;** PB: peripheral blood; CR: complete remission; PR: partial remission; CRS: cytokine release syndrome; ICANS: immune effector cell-associated neurotoxicity syndrome

^b^ N=12, numbers too small for multivariate analysis^c^ Analyses only includes patients without an event by 30 days post-infusion

**Table S8: Cases of non-relapse mortality in MCL patients after brexu-cel (n=12)**

| **Case** | **Age** | **Max grade**  **CRS/ICANS** | **ECOG pre-LD** | **Bulk >5cm pre-LD** | **Stage/LDH/EN sites pre-LD** | **Steroid dose /**  **duration** | **Neutrophil recovery** | **Day of death post infusion** | **Immune reconstitution (CD4/IgG)** | **Clinical history** |
| --- | --- | --- | --- | --- | --- | --- | --- | --- | --- | --- |
| **1** | 75 | 2/4 | 0 | No | 4/181/1 | NA/15 months | Yes | 479 | 0.00/On IVG (M14) | Progressive physical decline with cognitive dysfunction |
| **2** | 65 | 3/1 | 0 | No | 3/223/0 | 0 | Yes | 638 | 0.2/4.1  (M12) | SARS-CoV2 |
| **3** | 67 | 2/3 | 0 | No | 0/222/0 | 424mg/26 days | Yes (with stem cell top-up) | 224 | NA | Multiple bacterial infections |
| **4** | 68 | 2/3 | 1 | Yes | 4/384/3 | NA | No | 32 | NA | Sepsis (presumed bacterial) |
| **5** | 70 | 1/0 | 1 | No | 0/280/0 | 0 | Yes | 596 | NA/2.5  (M3) | SARS-CoV2 |
| **6** | 76 | 2/0 | 1 | Yes | 4/155/1 | 0 | Yes | 259 | 0.04/7.3  (M6) | SARS-CoV2 |
| **7** | 76 | 0/2 | 1 | Yes | 4/329/2 | 624/46 days | Yes | 306 | 0.03/On IVIG (M6) | Pulmonary fibrosis, on B/G pulmonary emphysema |
| **8** | 70 | 2/3 | 1 | Yes | 4/193/1 | 978/30 days | No | 82 | NA | Persistent cytopenias, declined further care |
| **9** | 64 | 1/0 | 1 | No | 3/111/1 | 0 | Yes | 308 | NA | Increasing frailty and transfusion requirements |
| **10** | 72 | 2/0 | 1 | NA | NA/264/NA | 0 | No | 38 | NA | Bacterial Sepsis |
| **11** | 78 | 5/1 | 2 | No | 4/687/2 | 338/6 days | No | 6 | NA | Suspected Grade 5 CRS |
| **12** | 66 | 2/2 | 0 | No | 2/216/0 | 120/5 days | No | 54 | NA | Bacterial Sepsis |

**Table S9: Progression-free survival (PFS) and overall survival (OS) from approval, harvest and infusion**

|  | **From approval (n=119)** | **From harvest (n=114)** | **From infusion (n=83)** |
| --- | --- | --- | --- |
| **PFS** |  |  |  |
| **Median months (95% CI)** | 11.4 (9.0-23.8) | 13.9 (9.3-N/A) | 21.0 (10.1-N/A) |
| **6-month PFS (95% CI)** | 62% (53-71) | 68% (58-76) | 82% (71-89) |
| **12-month PFS (95% CI)** | 47% (37-56) | 52% (40-62) | 62% (49-73) |
| **24-month PFS (95% CI)** | 35% (22-47) | 41% (27-54) | 48% (31-64) |
| **OS** |  |  |  |
| **Median months (95% CI)** | 14.9 (9.9-N/A) | 18.6 (11.2-N/A) | Not yet reached |
| **6-month OS (95% CI)** | 66% (56-74) | 72% (62-80) | 87% (76-93) |
| **12-month OS (95% CI)** | 54% (43-63) | 61% (50-70) | 74% (62-83) |
| **24-month OS (95% CI)** | 38% (25-50) | 44% (29-57) | 52% (33-68) |

**Table S10: Multivariable analysis of factors at submission associated with progression-free survival (PFS) and overall survival (OS)**

| **Variable ^a^** | **HR (95% CI), p-value**  **Complete cases (N=67)** | **HR (95% CI), p-value All available data (N=83)** |
| --- | --- | --- |
| **Progression-free survival** |  |  |
| Bulk (>5cm) at submission | 3.22 (1.34-7.71), p=0.009 | 3.16 (1.50-6.64), p=0.002 |
| Male | 7.06 (1.63-30.60), p=0.009 | 8.31 (1.97-35.13), p=0.004 |

**Overall Survival**

| Bulk (>5cm) at submission | 3.29 (1.21-8.95), p=0.02 | 3.21 (1.42-7.28), p=0.005 |
| --- | --- | --- |
| Male | 4.93 (1.10-22.04), p=0.04 | 6.58 (1.54-28.15), p=0.01 |

**^a^** Variables included. age 65+, sex, 3+ prior lines, LDH>ULN at submission, bulk >5cm at submission, 3+ EN sites at submission, Bendamustine before apheresis, ECOG at submission, stage at submission, sMIPI at submission

**Table S11 Multivariable analysis of factors pre-LD associated with progression-free survival (PFS) and overall survival (OS)**

| **Variable ^a^** | **HR (95% CI), p-value Complete cases (N=65)** | **HR (95% CI), p-value All available data (N=82)** |
| --- | --- | --- |
| **Progression-free survival** |  |  |
| Manufacture failure | 5.74 (1.77-18.63), p=0.004 | 2.75 (0.93-8.15), p=0.07 |
| Male | 22.40 (3.01-166.79), p=0.002 | 26.47 (3.80-184.47), p<0.001 |
| ECOG PS >1 pre-LD | 37.39 (5.45-256.79), p<0.001 | 41.85 (6.38-274.60), p<0.001 |

**Overall survival**

| Manufacture failure | 4.76 (1.50-15.08), p=0.008 | 2.73 (0.89-8.32), p=0.08 |
| --- | --- | --- |
| Male | 6.51 (1.31-32.25), p=0.02 | 8.64 (1.85-40.46), p=0.006 |
| ECOG PS >1 pre-LD | 8.35 (1.55-44.96), p=0.01 | 8.20 (1.64-40.88), p=0.01 |

^a^ Variables included: age 65+, sex, 3+ prior lines, bendamustine before first apheresis, ECOG pre-LD, weight pre-LD, LDH>ULN pre-LD, WCC at apheresis, lymphocyte count at apheresis >30, bulk >5cm pre-LD, 3+ EN sites pre-LD, response at time of infusion, manufacturing failure**.**

**Table S12: PFS and OS outcomes for those infused with/without manufacturing failure**

| **Variable** | **No manufacturing failure (N=75)** | | | **Manufacturing failure (N=8) ^a^** |
| --- | --- | --- | --- | --- |
| **Progression-free survival (PFS)** | | |  |  |
| Median PFS months (95% CI) | | 21.0 (10.1-NA) | | 7.4 (1.1-N/A) |
| 6-month PFS (95% CI) | 83% (72-90) | | | 75% (31-93) |
| 12-month PFS (95% CI) | 64% (50-75) | | | 50% (15-77) |
| 24-month PFS (95% CI | 49% (30-65) | | | N/A |
| **Overall survival (OS)** |  | | |  |
| Median OS (95% CI) | Not reached | | | 12.6 (2.7-N/A) |
| 6-month OS (95% CI) | 86% (76-93) | | | 88% (39-98) |
| 12-month OS (95% CI) | 76% (62-85) | | | 63% (23-86) |
| 24-month OS (95% CI) | 54% (37-70) | | | N/A |

^a^ Infused after 2^nd^ successful harvest and manufacture
